# Reassessment of intensive surveillance practices adopted for endometrial cancer survivors

**DOI:** 10.1186/s12905-022-01937-1

**Published:** 2022-08-23

**Authors:** Kazuto Nakamura, Yoshikazu Kitahara, Soichi Yamashita, Keiko Kigure, Ikuro Ito, Toshio Nishimura, Anri Azuma, Tatsuya Kanuma

**Affiliations:** 1 Department of Gynecology, Gunma Prefectural Cancer Center, Ota, Japan; 2 Department of Obstetrics and Gynecology, Gunma University, Maebashi, Japan; 3 Department of Obstetrics and Gynecology, Takasaki General Medical Center, Takasaki, Japan

**Keywords:** Endometrial cancer, Surveillance, Local recurrence

## Abstract

**Background:**

In Japan, 17,000 women are newly diagnosed with endometrial cancer in 2018. The healthcare insurance policy in Japan provides more intensive patient surveillance compared with the United States and European countries. The aim of this study was to retrospectively analyze data, including surveillance methods, recurrence sites, salvage therapy, and survival period after recurrence, to consider the benefits of surveillance for patients with endometrial cancer.

**Methods:**

Between January 2009 and December 2015, the medical records of patients who were initially diagnosed with the International Federation of Gynecology and Obstetrics stage I–IV endometrial cancer and treated were enrolled in this retrospective study. Only patients with stage IV cancer with peritoneal dissemination were included. Within the first 2 years, the included patients underwent tumor marker tests, Papanicolaou smear test every 1–3-months, and imaging analysis at 6–12- month intervals. Until 4 years, the patients underwent regular surveys every 4 months and imaging analysis annually. Subsequently, the patients received regular surveys every 6 -to 12-months. Results.

Among 847 patients, 88 experienced recurrence, and their clinicopathological data were statistically analyzed. The recurrence site was not associated with the initial treatment method or histology. Among the patients with recurrence, 75% were asymptomatic. Univariate analysis demonstrated that time to recurrence and local recurrence were significant factors for survival outcomes, whereas multivariate analysis indicated that only local recurrence was a significant factor. In patients with distant metastasis, neither symptomatic nor asymptomatic recurrence showed a significant difference in survival.

**Conclusions:**

In this retrospective study, an intensive surveillance protocol did not benefit patients with endometrial cancer. Thus, we hypothesize that the characterization of tumors by emerging technologies that can precisely predict the nature of the tumor will help tailor individualized and efficient surveillance programs. In addition, the ideal salvage therapy needs to be developed to benefit patients after recurrence.

## Background

Endometrial cancer is the most common gynecological cancer in Japan. Approximately 17,000 patients were newly diagnosed in 2018, and 80% of the patients were categorized as stage I and II. Thus, the majority of patients are expected to have a favorable prognosis; however, some women experience recurrent tumors. A systematic analysis by Fung-Kee-Fung et al. [1] demonstrated an overall risk of endometrial cancer recurrence of 13%, which was 3% in low-risk patients. To date, numerous studies have examined prognostic factors following recurrence, such as initial stage [2], tumor histology and grade, CA125 level at recurrence [3], metastatic site [4], symptomatic or asymptomatic recurrence [5, 6], and time to recurrence from initial treatment [7, 8]. In addition to the clinicopathological factors mentioned above, The Cancer Genome Atlas (TCGA) Research Network has demonstrated four prognostic categories of molecular signatures in endometrial cancer: mutation in polymerase-ε (POLE), (best prognosis),; mismatch repair protein deficiency, (intermediate prognosis),; Tumor Protein 53 mutation, (worst prognosis),; copy-number low, (good to intermediate prognosis) [9]. Thus, the European Society of Gynecological Oncology, European Society for Radiotherapy and Oncology, and European Society of Pathology have jointly proposed a new management strategy for endometrial cancer patients, utilizing histological and molecular features [10].

Post-initial treatment surveillance is intended for early detection of recurrence and to provide the appropriate treatment and psychological support for the patient. However, there are no definitive surveillance guidelines that allow physicians to adopt variable surveillance protocols, as most studies are based on retrospective analyses, and limited data have been incorporated into the current guidelines for post-treatment surveillance.

In Japan, nearly all patients are covered by public health insurance, which is applicable to the majority of medical charges, allowing physicians to perform intensive surveillance using the Papanicolaou (Pap) smear test, measurement of CA-125 levels at every visit, and periodic imaging analysis, such as computed tomography (CT), magnetic resonance imaging, and positron emission tomography (PET) scan for endometrial cancer patients even without any symptoms. In contrast with the Society of Gynecologic Oncologists recommendations [11], the Japan Society of Gynecologic Oncology recommends these tests every 1-to 3- months for the first 1- to 3- years and every 6 months for the 4th and 5th years after treatment [12]. There is an increasing number of endometrial cancer patients in Japan, with an incidence of 26.3/100,000 newly diagnosed women in 2018, which was more than double that in 2000. Thus, there is a need to establish appropriate surveillance protocols that provides clinical and cost-effective practices for detecting recurrence and improving survival outcomes.

In this study, we aimed to re-examine intensive surveillance protocols by retrospectively analyzing data, including surveillance methods to detect recurrence, recurrence sites, and survival periods after recurrence, to consider surveillance benefits for patients and cost-effective practices.

## Methods

### Patients

After receiving institutional review board approval from the Ethics Committee of the Gunma Prefectural Cancer Center (approval # 405-31,012), a multicenter study was conducted. Three participating institutions belonging to the Gunma Medical Local Society were included in this study: Gunma Prefectural Cancer Center, Gunma University, and Takasaki General Medical Center. The study protocol was approved by the Gunma University Hospital Clinical Research Review Board and the Ethics Review Committee of the National Hospital Organization Takasaki General Medical Center.

Under the ethical guidelines for medical and health research involving human subjects in Japan, informed consent is not required for medical study that uses only medical records without the use of human samples. Thus, informed consent was not obtained from the participants in this study; instead, all participants were given the right to withdraw their consent for the use of the data with an opt-out method.

The medical records of patients with endometrial cancer whose cancers were diagnosed according to the International Federation of Gynecology and Obstetrics (FIGO) stage I–IV disease and treated between 2009 and 2015 were obtained from three institutions. Stage IV patients, with only peritoneal dissemination, were included in this study.

### Surveillance protocol

The basic follow-up schedule was conducted every 1–3 months for the first 2 years, every 4 months for the next 2–4 years, and every 6 months thereafter. Pelvic examination, Pap smear, transvaginal ultrasound scanning, and CA125 measurement were performed at every visit, and imaging tests, such as CT and PET, were conducted every 6–12 months. Demographic data, FIGO stage, histology, initial therapy, method of diagnosis for recurrent tumors, treatment after recurrence, and survival period after recurrence were obtained from the patients’ medical records. Intervals between visits before the diagnosis of recurrence were stratified into 1, 2, and > 3 months.

### Statistical analysis

The chi-squared test was used to analyze the association between the recurrence site and initial treatment or histological endometrial cancer. The correlation between the site of recurrence and the method of diagnosis of recurrence was analyzed using correspondence analysis. Cox regression analysis was performed to calculate hazard ratios and 95% confidence intervals (95% CIs) for each factor (FIGO stage, histological type, initial treatment, time to recurrence, recurrence site, and diagnostic method of recurrence) that may be associated with overall survival after recurrence. In addition, multivariate Cox regression analysis was performed using a stepwise variable selection method for recurrence sites that showed statistically significant differences in the univariate analysis. For patients in the local recurrence group, we performed a more detailed analysis using the Mann–Whitney *U* test for age and chi-squared test for initial staging, histology, time to recurrence, hospital history, subjective symptoms, and treatment after recurrence. The survival curves for overall survival after recurrence with and without subjective symptoms were calculated using the Kaplan–Meier method and log-rank test. The Mann–Whitney *U* tests were two-tailed, and the chi-squared test was performed using Fisher's exact test. Statistical significance was set at p < 0.05. All statistical analyses　were performed using SAS ver. 9.4 (SAS Institute Inc, Cary, NC, USA).

## Results

### Patient characteristics

During the study period, 847 patients were treated for endometrial carcinoma at three institutions in Gunma Prefecture, Japan. A total of 88 patients who developed recurrence were enrolled in this retrospective study. Sixty-five percent and 79% of patients experienced recurrence within 2 and 3 years, respectively, and the overall risk of recurrence was 10.4%. The risk of recurrence for each stage was as follows: 5.2% in stage I, 8.2% in stage II, 24.2% in stage III, and 27.1% in stage IV. The basic characteristics of the patients with recurrent disease, including medians and ranges for age, FIGO stage, endometrial cancer histology, initial treatment, recurrence site, and diagnostic method of recurrence, are shown in Table 1. At the initial diagnosis, 30 patients (34.1%) were diagnosed with stage I, 52 patients (59.1%) had endometrioid G1-2 tumors, and 53 patients (60.2%) underwent lymph node (LN) (pelvic or pelvic and para-aortic) resection. Sixty patients (68.2%) received chemotherapy and four received neoadjuvant chemotherapy. None of the patients in this study received adjuvant radiation therapy after the primary surgery.

**Table 1:** Patients characteristics at initial treatment and at recurrence

Characteristics	Patients	n = 88
Initial cancer		
Age	32–82 (63)	
FIGO stage		
I	30	(34.1)
II	4	(4.5)
III	38	(43.2)
IV	16	(18.2)
Histology		
Eendometrioid G1-G2	52	(59.1)
Endometrioid G3	15	(17.0)
Serous	5	(5.7)
Others	16	(18.2)
Initial treatment		
Operation		
ATH + BSO	19	(21.6)
ATH + BSO + PLA biopsy	12	(13.6)
ATH + BSO + PLA	31	(35.2)
ATH + BSO + PLA + PAN	22	(25.0)
NAC + ATH	4	(4.5)
Chemotherapy		
No	28	(31.8)
Yes	60	(68.2)
At recurrence		
Follow up interval		
1 month	44	(50.0)
2 months	20	(22.7)
3 > months	24	(27.3)
Recurrence site		
Local	17	(19.3)
Pelvic and para-aortic LN	13	(14.8)
Peritoneal dissemination	13	(14.8)
Distant metastasis	45	(51.1)
Method of diagnosis		
Symptom	22	(25.0)
Tumor marker	16	(18.2)
Imaging analysis	37	(42.0)
Outpatient exam	13	(14.8)

Range (median); ATH: abdominal hysterectomy; BSO: bilateral salpingo oophorectomy; PLA: pelvic lymph adenectomy; PAN: para-aortic lymphadenectomy; NAC: neoadjuvant chemotherapy; LN: lymphnode; Out patient exam: pelvic examination, pap smear, and transvaginal ultrasoound scannning

### Association between recurrence site and diagnostic modality

At recurrence, 64 patients (72.7%) were surveyed within a 2-month interval. Local recurrence occurred in 17 patients (19.3%), and clinical symptoms were observed in 22 patients (25.0%). There was no relationship between the initial treatment procedure, histology, FIGO stage, and recurrence site (Table 2).

**Table 2:** Recurrence site correlated with initial treatment, histology, and FIGO stage

	Local	Regional LN	Peritoneal dissemination	Distant metastasis	*p-*value*
Initial treatment					0.606
ATH + BSO	1	4	4	10	
ATH + BSO + PLA	9	3	5	14	
ATH + BSO + PLA biopsy	3	2	2	5	
ATH + BSO + PLA + PAN	4	2	2	14	
NAC + ATH	0	2	0	2	
Histology					0.098
Endometrioid G1/G2	13	10	7	22	
Endometrioid G3	3	1	1	10	
serous	0	1	2	2	
others	1	1	3	11	
FIGO Stage					0.650
I	7	3	7	13	
II	1	1	0	2	
III	8	7	4	19	
IV	1	2	2	11	

ATH: abdominal hysterectomy; BSO: bilateral salpingo oophorectomy; PLA: pelvic lymph adenectomy; PAN: para-aortic lymphadenectomy; NAC: neoadjuvant chemotherapy; LN: lymphnode

*Chi-square test was used

**Fig. 1:**
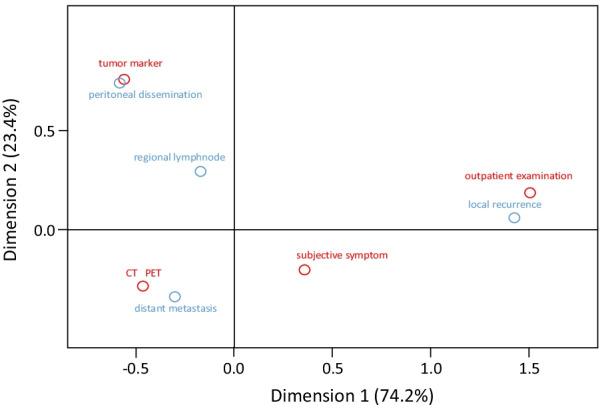
Correspondence analysis demonstrating the association of diagnostic method with recurrence site CT, computed tomography; PET, positron emission tomography

We performed a correspondence analysis to identify the association between recurrence sites and diagnostic modalities for detecting recurrence (Fig. 1). Outpatient examinations (physical examination, Pap smear, and transvaginal ultrasound scanning) were correlated with local recurrence, and tumor marker CA125 level was associated with peritoneal dissemination. Imaging analysis (CT and PET) correlated with distant metastasis, whereas subjective symptoms were not related to any recurrence site. In fact, local recurrence was mainly diagnosed according to subjective symptoms (7/17, 41.2%) or Pap smear results (9/17, 52.9%), and distant metastasis was detected by imaging analysis (29/45, 64.4%) and tumor marker (5/45, 11.1%). Meanwhile, dissemination was elicited by CA125 measurement (7/13, 53.8%) and imaging analysis (4/13, 30.8%).

### Survival outcomes after recurrence

**Fig. 2:**
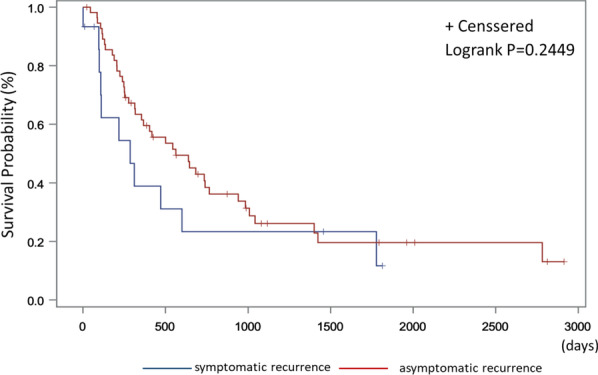
Overall survival after symptomatic or asymptomatic recurrence in patients with distant metastasis

The Kaplan–Meier curve (Fig. 2) showed that patients with asymptomatic distant metastasis detected by routine imaging analysis did not have better survival than those with particular symptoms, which led to unscheduled imaging analyses (hazard ratio, 0.672; 95% CI, 0.343–1.318; p = 0.248).

The hazard ratios and 95% CIs for survival after recurrence were calculated by Cox regression analysis (Table 3), which revealed no significant difference for FIGO stage, histology, initial treatment, and diagnostic method at the end of the study observation period; however, the time to recurrence was a significant factor. Local recurrence demonstrated a significantly better prognosis for pelvic and para-aortic LN and distant metastases. Multivariate analysis of the recurrence site revealed that local recurrence was the only prognostic factor.

**Table 3:** Univariate and multivariate analysis for survival outcomes after recurrence

	Univariate		Multivariate	
	Hazard ratio [95% CI]	*p*-value*	Hazard ratio [95% CI]	*p*-value*
FIGO stage				
I	ref			
II	1.736 [0.498–6.048]	0.387		
III	1.271 [0.678–2.385]	0.454		
IV	1.767 [0.845–3.691]	0.130		
Histology				
Endometrioid G1/G2	ref			
Endometrioid G3	1.151 [0.550–2.408]	0.708		
Serous	0.906 [0.314–2.612]	0.885		
Other	1.792 [0.791–4.055]	0.162		
Initial treatment				
ATH + BSO	ref			
ATH + BSO + PLA biopsy	0.678 [0.280–1.638]	0.388		
ATH + BSO + PLA	0.659 [0.327–1.329]	0.244		
ATH + BSO + PLA + PAN	0.596 [0.275–1.288]	0.188		
NAC + ATH + BSO	0.830 [0.235–2.928]	0.772		
Time to recurrence	0.999 [0.0998–1.000]	0.008		
Recurrence site				
Local	ref		ref	
Pelvic and para-aortic LN	5.836 [2.143–15.892]	0.001	5.311 [1.866–15.114]	0.002
Peritoneal dissemination	3.088 [1.061–8.984]	0.039	2.699 [0.910–8.004]	0.073
Distant metastasis	4.100 [1.6961–9.911]	0.002	3.876 [1.542–9.743]	0.004
Diagnosis method at recurrence				
Outpatient exam	ref			
Symptom	1.042 [0.429–2.529]	0.928		
Tumor marker	1.830 [0.797–4.353]	0.172		
Imaging analysis	1.385 [0.640–2.999]	0.409		

ATH: abdominal hysterectomy; BSO: bilateral salpingo oophorectomy; PLA: pelvic lymphadenectomy; PAN: para-aortic lymphadenectomy; NAC: neoadjuvant chemotherapy; LN: lymphnode; Out patient exam: pelvic examination, pap smear, and transvaginal ultrasoound scannning

*Cox regression analysis was used

### Outcomes of salvage therapy

We investigated the salvage therapy outcomes for local recurrence (Table 4). There were no significant differences for initial stage, histology, scheduled visit, and symptoms at recurrence. However, the time to recurrence was significantly longer in the no-recurrence group (1,099 days [27–118]) than in the death**-**after**-**recurrence group (226 days [161–1,000]), and was also a significant factor for salvage therapy outcomes for local recurrence.

**Table 4:** Outcomes after salvage therapy for local recurrence

	No recurrence	Die after recurrence	*p-*value
Age	58 [41–78]	67 [51–79]	0.086**
Initail stage			0.091*
1a	4	0	
1b	0	3	
2	0	1	
3a	3	0	
3c	4	1	
4b	0	1	
Histology			0.655*
Endometrioid G1-2	8	5	
Endometrioid G3	2	1	
Carcinosarcoma	1	0	
Time to recurrence (day)	1,099 [27–2,118]	226 [167–1,000]	0.021**
Shcedulled visit at recurrence			0.091*
No	4	0	
Yes	7	6	
Symptom at recurrence			0.064*
No	4	5	
Yes	7	1	
Treatment after recurrence			0.434*
Supprotive care	0	1	
Radiation	10	4	
Chemotherapy	1	1	

*Chi-square test was used; **U-test was used

## Discussion

The majority of patients with recurrent endometrial cancer have a poor prognosis, although some specific cases demonstrate long-term survival after recurrence. Post-treatment surveillance is expected to detect recurrent tumors at a very early stage, and these tumors can be completely cured with multidisciplinary therapy. To our knowledge, this study analyzed the most intensive surveillance methods available in daily clinical practice under the public health insurance coverage in Japan. In the present study, the time to first recurrence and local recurrence were significant predictive factors for better prognosis after recurrence. However, although 75% of recurrences were diagnosed as asymptomatic, the intensive surveillance protocol did not substantially improve post-recurrence outcomes.

Patients with cancer desire survival benefits with routine surveillance to detect early recurrence. In this study, nearly all patients were observed by gynecologic oncologists instead of being transitioned back to primary care providers. According to the intensive surveillance protocol analyzed in this study, 72.7% of recurrences were diagnosed within the 2-month follow-up interval, and 75.0% of patients had no symptoms (Table 1). Ueda et al. showed that asymptomatic recurrence had a better prognosis without statistical significance [6], and an Italian multicenter retrospective analysis also supported the benefit of diagnosing asymptomatic recurrence [13], with local recurrence rates of 55.2% and 40.6%, respectively, in contrast to our finding of a local recurrence rate of 19.3%. A low local recurrence rate might be the reason asymptomatic recurrence did not have a better prognosis in our study, as local recurrence is a better prognostic factor (Table 3).

Correspondence analysis between the diagnostic method and recurrence site indicated that tumor marker levels were associated with peritoneal dissemination, and imaging analysis was related to distant metastasis (Fig. 1). Similar to ovarian cancer, CA125 has been used as a marker of endometrial cancer recurrence. Similar to other reports [1, 4], patients with asymptomatic recurrence diagnosed by elevated CA125 levels had peritoneal dissemination (8 patients, 50%) or distant metastasis (4 patients, 25%); however, 13 of 16 patients eventually died of recurrence in this study. Thus, our results are consistent with the recommendations of the 2021 version of the National Comprehensive Cancer Network guidelines, which do not recommend CA125 for routine surveillance, suggesting that CA125 measurements should only be used in specific patients with advanced disease, serous carcinoma, or pre-elevated CA125 levels before treatment [14].

Imaging analysis is commonly used to detect recurrence. In this study, 45 patients (51.1%) were diagnosed with distant metastasis (Table 1). Several reports have described the predictive factors for the risk of distant metastasis. Tumor grade, deep myometrial invasion, and extrauterine disease are independent risk factors for distant metastasis [15–17]. Although the recurrence site was not related to the initial treatment method, histology, or FIGO stage, high-grade endometrioid and non-endometrioid tumors indicated a predisposition for distant metastasis (Table 2). An important purpose of routine imaging analysis is the detection of asymptomatic distant metastasis to determine the utility of treatment for recurrence since CT and PET appear to be more sensitive for identifying recurrent lesions [18, 19]. In this study, 33 of 45 patients with distant metastasis were diagnosed as asymptomatic, and only 4 patients did not have subsequent recurrence after salvage therapy. Three of four patients with no further recurrence benefited from imaging analysis that detected a single lesion in the lung, which could be treated, similar to other reports [20, 21]. In addition, asymptomatic distant metastasis detected by imaging analysis did not show a significant difference in overall survival after recurrence (Fig. 2). Owing to the lack of supportive evidence for imaging analysis, its purpose should be reconsidered according to the Society of Gynecologic Oncologists surveillance recommendations, which advise not to use CT/PET scans without suspected symptoms [11].

Consistent with other studies [22, 23], local recurrence demonstrated better survival after recurrence in both univariate and multivariate analyses in this study compared with pelvic and para-aortic LN metastasis, peritoneal dissemination, and distant metastasis (Table 3). The efficacy of cytological screening for vaginal cuff recurrence has been vigorously discussed, and retrospective studies have demonstrated a low rate of detection (< 6.8%) [24] and low cost-effectiveness in asymptomatic recurrence [25]. Thus, the Society of Gynecologic Oncologists and European Society for Medical Oncology guidelines [26] do not recommend routine cytologic evaluation of the vaginal cuff. Adjuvant brachytherapy with or without pelvic radiation has been widely adopted in the United States and European countries, resulting in the successful reduction of local recurrence. In contrast, following the results of the Gynecologic Oncology Group 122 [27] and Japanese Gynecologic Oncology Group 2033 studies [28], chemotherapy has mainly been utilized as a postoperative adjuvant therapy in Japan [29]. Adjuvant radiation therapy after surgery was adopted at a rate of 12.8% in 2013. This result differentiates the significance of Pap smears in Japan from that in other countries. In this study, 9 of 17 patients with asymptomatic local recurrence were diagnosed using Pap smear results; however, 5 of the 9 asymptomatic patients eventually died. Moreover, as identified in this study, previous studies also found that time to recurrence is a prognostic factor (Table 4) [30, 31]. Even if the local recurrence is asymptomatic, tumor aggressiveness may predict survival after recurrence. Following the analysis in TCGA in 2013 [9], Wortman et al. explained that specific molecular signatures in endometrial cancer are associated with recurrence patterns [32]. Molecular features in mainly high-intermediate risk patients from the PORTEC-1 and -2 trials revealed that marked lymphovasucular space involvement, p53-mutation, and L1CAM expression were correlated with pelvic recurrence and distant metastasis [33]. Moreover, even in a small number of cases, patients with POLE mutations who received no adjuvant therapy had a favorable prognosis [34]. Characterization of the nature of the tumor by assessing its molecular and clinicopathological features may help tailor specific individualized surveillance protocols in terms of clinical and economic benefits.

This study was conducted using multicenter data to decrease patient selection bias. However, there were some limitations, including the retrospective nature of the study and small sample size for the stratified group analysis based on prognostic factors. The results of our study require further validation by future studies with larger cohorts and randomized controlled trials.

## Conclusions

Although our study indicated that patients with local recurrence with a long time to recurrence had a better prognosis, our intensive surveillance protocol did not benefit patients with asymptomatic recurrence to a large extent. However, we believe that surveillance provides psychological support and effective control of symptoms caused by recurrence in survivors. Further development of treatment modalities and therapeutic approaches for recurrent endometrial cancer are required to establish effective strategies.

## Data Availability

Not applicable.

## Abbreviations

Pap smear test: Papanicolaou smear test
CT: Computed tomography
PET: Positron emission tomography
FIGO: International Federation of Gynecology and Obstetrics
LN: Lymphnode
TCGA: The Cancer Genome Atlas
LN: Lymph node
